# Automated tracking of label-free cells with enhanced recognition of whole tracks

**DOI:** 10.1038/s41598-019-39725-x

**Published:** 2019-03-01

**Authors:** Naim Al-Zaben, Anna Medyukhina, Stefanie Dietrich, Alessandra Marolda, Kerstin Hünniger, Oliver Kurzai, Marc Thilo Figge

**Affiliations:** 1 Applied Systems Biology, Leibniz Institute for Natural Product Research and Infection Biology – Hans Knöll Institute (HKI), Jena, Germany; 2 Faculty of Biological Sciences, Friedrich Schiller University Jena, Jena, Germany; 3 Fungal Septomics, Leibniz Institute for Natural Product Research and Infection Biology – Hans Knöll Institute (HKI), Jena, Germany; 4 Institute of Hygiene and Microbiology, University of Würzburg, Würzburg, Germany; 5 Center for Sepsis Control and Care (CSCC), Jena University Hospital, Jena, Germany

## Abstract

Migration and interactions of immune cells are routinely studied by time-lapse microscopy of *in vitro* migration and confrontation assays. To objectively quantify the dynamic behavior of cells, software tools for automated cell tracking can be applied. However, many existing tracking algorithms recognize only rather short fragments of a whole cell track and rely on cell staining to enhance cell segmentation. While our previously developed segmentation approach enables tracking of label-free cells, it still suffers from frequently recognizing only short track fragments. In this study, we identify sources of track fragmentation and provide solutions to obtain longer cell tracks. This is achieved by improving the detection of low-contrast cells and by optimizing the value of the gap size parameter, which defines the number of missing cell positions between track fragments that is accepted for still connecting them into one track. We find that the enhanced track recognition increases the average length of cell tracks up to 2.2-fold. Recognizing cell tracks as a whole will enable studying and quantifying more complex patterns of cell behavior, e.g. switches in migration mode or dependence of the phagocytosis efficiency on the number and type of preceding interactions. Such quantitative analyses will improve our understanding of how immune cells interact and function in health and disease.

## Introduction

Proper functioning of the immune system relies on adequate behavior of individual immune cells. A powerful way to study how immune cells migrate and interact is by time-lapse microscopy of *in vitro* migration and confrontation assays, where immune cells either migrate alone on an imaging dish or are confronted with pathogens^1^. The relevance of *in vitro* assays was exemplified in our recent study of monocytes and polymorphonuclear neutrophils (PMN) phagocytosing two fungal species: *Candida albicans* and *Candida glabrata*^2^. In an *in vitro* assay we showed that *C. glabrata* is more efficiently recognized by monocytes, while PMN prefer to uptake *C. albicans* – a finding that we subsequently confirmed in a human whole-blood infection model^2^. Thus*, in vitro* assays provide a relatively simple setting to generate new hypotheses that can be then validated under more realistic physiological conditions.

To get the most of this powerful method, *in vitro* assays should be combined with automated image analysis and tracking: To objectively characterize cell behavior, the assays must be repeated many times, which inevitably generates large amounts of data. This is especially relevant when analyzing rare events that only occur in a few percent of all cell interactions. For example, we recently observed that PMN occasionally release phagocytosed *C. glabrata* cells after killing them intracellularly^3^, which may enable the pathogens to be subsequently taken up and processed by professional antigen presenting cells. To scrutinize the details of this “dumping” process and its implications for antigen presenting cells, we have to analyze large amounts of video data. Such analysis is too tedious to be performed manually and requires automated image segmentation and tracking.

**Figure 1:**
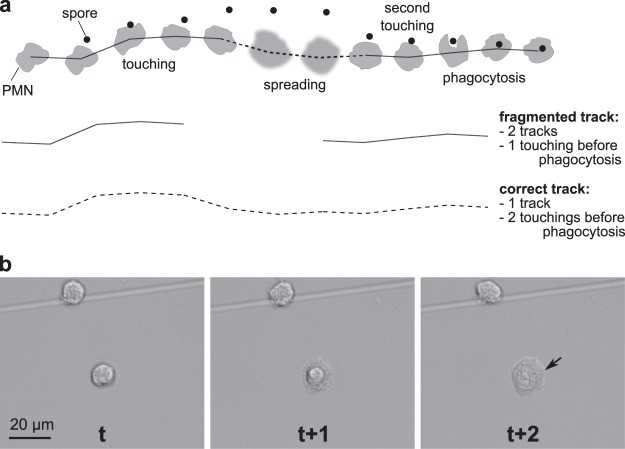
Track fragmentation due to transient spreading. (**a**) A cell track may become fragmented when the cell spreads and escapes detection by the tracking algorithm; the algorithm assigns the cell to two separate tracks, and incorrectly estimates the number of touching events before phagocytosis. (**b**) Example of a spreading human polymorphonuclear neutrophil (PMN) (indicated by arrow). PMN were followed over a time period of one hour using bright-field microscopy and images were taken at six frames per minute.

Unfortunately, many existing cell tracking approaches (for an overview, see^4–6^) suffer from two main weaknesses: they heavily rely on staining of the visualized cells and they produce rather short cell trajectories. And while motility of murine cells can be successfully studied using numerous available reporter mice^7,8^, fluorescent staining of human immune cells may alter their behavior and provoke cell death. To enable the quantitative motility analysis of label-free human cells, we previously developed algorithm for migration and interaction tracking (AMIT)^9,10^, which allowed tracking of label-free immune cells in bright-field microscopy videos. However, a continuous tracking of individual cells for as long as possible still remained unresolved: both our previous algorithm and many other tracking approaches^11^ detect rather short fragmented tracks. Because fragmentation of cell tracks may obscure complex patterns in cell behavior, it is of utmost importance to identify cell tracks uninterrupted throughout the entire time course. If cell tracks are identified only as fragmented tracklets, correlations and rare functional relationships between time-separated events may be entirely missed (see e.g. Fig. 1a). While the observation time of each cell track is unavoidably limited by the microscope’s finite field of view, we should strive to optimize tracking algorithms to detect complete cell tracks within the given field of view in order to fully exploit the available data basis and acquire statistically sound results.

With the goal to detect complete cell tracks we therefore searched for the sources of track fragmentation and for strategies to reduce it. We visually examined the AMIT tracking results and identified three possible reasons why cell tracks become fragmented: (1) tracklets are mismatched when resolving clusters of interacting cells, (2) tracks are interrupted on cells leaving the focal plane by transient spreading^12,13^ (see Fig. 1b), and (3) automated tracking employs too low values of the gap size – a parameter that defines the number of allowed missing time steps between tracklets when connecting them to longer tracks. In this study, we investigate how and whether these issues contribute to track fragmentation and introduce an improved second version of AMIT.

## Methods

### AMIT-v1: First version of an algorithm for tracking of label-free cells

**Figure 2:**
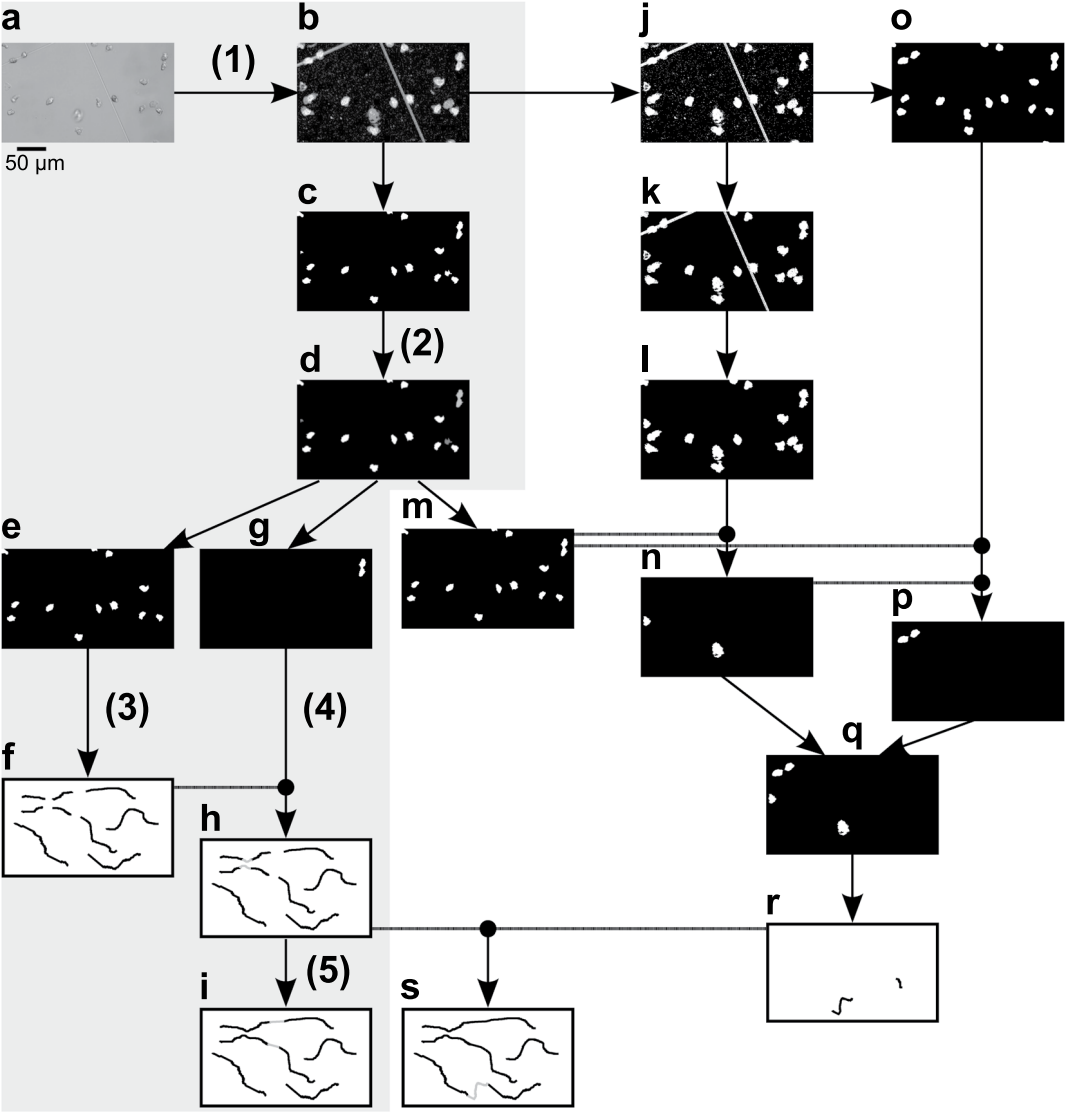
Workflow of AMIT-v1 (shaded area) and AMITv2 (the full scheme). Numbers in brackets correspond to the five steps of AMIT-v1. Input image (**a**) is segmented with a Gaussian mixture model (**b**) into background (black), static objects (gray) and mobile objects (white). Mobile objects (**c**) are further segmented based on object area (**d**) into noise (dark gray), single cells (white) and cell clusters (light gray). Single cells (**e**) are tracked by overlap (**f**), and cell clusters (**g**) are split by ellipse fitting and added to the single cell tracklets (**h**). All tracklets are combined into the final AMIT-v1 tracks by graph optimization (**i**). To extract spreading cells, mobile and static objects are combined into one mask (**j**), noise is removed based on area (**k**), and grid lines of the imaging dish are removed via ellipse fitting (**l**). Remaining objects (**l**) are compared with the combined mask of static and mobile objects from AMIT-v1 (**m**), and only newly detected objects are kept (**n**). To disconnect spreading cells from the grid lines, (**j**) is processed by morphological opening and closing (**o**), and cells that do not overlap with any object from (**m**) or (**n**) are isolated (**p**). Images (**n**) and (**p**) are combined into the final spreading cell image (**q**). Spreading cells are tracked (**r**), and included in the graph optimization step to obtain final AMIT-v2 tracks (**s**). Panels (**f,h,i,r,s**) show a schematic representation of tracks, while all the other panels show different processing steps of the real image from (**a**).

We previously developed a framework that tracks both cell migration paths and cell interactions and is hence named “algorithm for migration and interaction tracking” (AMIT)^9,10^. This first version of AMIT – which is here referred to as AMIT-v1 – consists of two parts: the first part tracks non-rigid label-free cells (e.g. PMN) without assuming any specific migration model^9^, while the second part tracks fluorescently-labeled cells (e.g. pathogenic fungus) and identifies and records all interactions that the cells experience during the course of the study^10^. The weakness of AMIT-v1 is that it often fails to detect very low-contrast cells which mainly occur due to transient spreading (Fig. 1b). Therefore we aim to improve the segmentation and tracking of such cells by optimizing the tracking part of the framework. This part of AMIT-v1 comprises five main steps^9^, which are illustrated in the shaded part of Fig. 2 and briefly summarized in what follows:

1. Input images (Fig. 2a) are segmented in the space of spatio-temporal intensity variances with a Gaussian Mixture Model (GMM) to distinguish between background, moving objects and static objects. To this end, the variances of pixel intensities are computed in the spatial and temporal neighborhood, and each pixel is assigned to one of the three classes: background with relatively low spatial and low temporal variances (black color in Fig. 2b), static objects with a relatively low temporal or spatial variance (gray color), and mobile objects with relatively high spatial and high temporal variance (white color). Thus, moving cells are assigned to the latter class (Fig. 2c).
2. Single cells are separated from noise and cell clusters by a second GMM (Fig. 2d). This GMM is fitted to the distribution of areas of all objects that were detected in the previous step. We imposed the condition on the GMM to fit three classes (noise, single cells, cell clusters), where the corresponding separation criteria between sizes of objects are learned from the data in an unsupervised manner.
3. Single cells (Fig. 2e) are tracked across all time frames based on the overlap between two cells in consecutive time frames. This generates a set of tracklets (Fig. 2f).
4. Cell clusters (Fig. 2g) are split into their constituent cells via ellipse fitting. The number of constituent cells is determined from the cluster area and the number of overlapping single cells in the preceding and/or subsequent time frames. The tracklets of the extracted cells are added to the corresponding tracklets of single-cells (Fig. 2h).
5. Cell tracklets are combined into longer tracks (Fig. 2i) by optimizing a distance graph. Tracklets can be connected even if several time steps between them are missing. The number of allowed missing time steps is user-defined and referred to as the gap size.

The performance of AMIT-v1 was evaluated^9,10^ with regard to the manual ground truth tracking and compared to the performance of two further software packages, *i.e*. the MOSAIC toolbox^14^ and the DYNAMIK toolbox^15^. AMIT-v1 was superior to both tools in distinguishing the low contrast cells from the background, resulting in lower track false positive and track fragmentation errors. The main objective of the present study is to further decrease track fragmentation and enhance recognition of whole tracks.

### AMIT-v2: Second version of the tracking algorithm with improved detection of spreading cells

AMIT-v1 relies on high spatial and temporal variances to detect moving objects and therefore often fails to detect low-contrast spreading cells (Fig. 1b). If a cell is lost for a period longer than the user-defined gap size, the track of the cell becomes fragmented.

In AMIT-v2, we reduce track fragmentation by recovering lost connections between tracklets that are induced by transient spreading of cells^12,13^. Spreading cells have low intensity contrast and hence relatively low spatial variance and therefore often erroneously end up in the class of static objects. Most of the objects in this class, however, are parts of other cells, noise, and grid lines of the cell culture imaging dish (gray regions in Fig. 2b). Imaging dishes with grid lines were used for focusing purposes as well as for being able to correct for potential artefacts due to movements of the dish. The latter were however not observed and hence no corrections of artefacts were necessary.

To distinguish spreading cells from other irrelevant regions, we developed the following segmentation procedure (unshaded part of Fig. 2):

First, we separate cells from noise and grid lines and identify candidate spreading cells. For this, we combine the static and mobile objects from Fig. 2b into one binary mask (Fig. 2j). We segment this mask with an area-based GMM – similarly to step (2) of AMIT-v1 – and discard noise while keeping single cells and cell clusters (Fig. 2k). To remove elongated shapes, we fit an ellipse to each object and discard objects with a ratio of minor to major axis smaller than 25%. Finally, we close gaps inside objects via a binary hole filling (Fig. 2l).

Cells extracted by this procedure tend to be over-detected. Therefore, we use AMIT-v1 segmentation results for all already detected not spreading cells, and isolate only those cells that were newly segmented. For this, we compare the results from Fig. 2l to the binary mask with single cells and cell clusters detected by AMIT-v1 (Fig. 2m) and select only those objects from Fig. 2l that do not overlap with any object from Fig. 2m. The result is shown in Fig. 2n.

Next, we recover those spreading cells that overlap with grid lines of the cell culture imaging dish and were discarded during ellipse fitting. To disconnect such cells from the grid lines, we process Fig. 2j with morphological opening and closing using a 15 × 15 pixels square as a kernel (Fig. 2o). To separate spreading cells from previously detected cells, we select only those objects from 2o that do not overlap with any object from Fig. 2m and Fig. 2n. The result is shown in Fig. 2p. Figure 2n,p are then combined into an image including all spreading cells (Fig. 2q).

Finally, we use the recovered spreading cells to prolong and connect the tracks from AMIT-v1. Similarly to step (3) of AMIT-v1, we track spreading cells by overlap to obtain spreading cell tracklets (Fig. 2r). We then incorporate these tracklets into the graph optimization procedure (step 5 of AMIT-v1) and combine them with other tracklets of AMIT-v1 (Fig. 2s). We discard tracklets that do not connect or prolong other AMIT-v1 tracks to avoid noise that is occasionally extracted from the static objects class.

### Evaluating performance of tracking

To compare the performance of AMIT-v1 and AMIT-v2, we compute the track fragmentation error (TFE) and track merging error (TME) used previously^9,10^. The errors are defined as follows:

1 $$TFE=\,\frac{nST \sim GT}{nGT},$$

2 $$TME=\frac{nST \sim GT}{nST}.$$

Here, ST refers to system tracks (i.e., tracks detected by the algorithm), and GT refers to ground truth tracks (i.e., tracks detected manually); $nGT$ is the number of ground truth tracks, $nST$ is the number of system tracks, and $nST \sim GT$ is the number of ST-GT matches/associations (see, e.g., Fig. S1). Thus, TFE refers to the average number of ST-fragments per GT, while TME denotes the average number of merged fragments from different GT per one ST. A perfect tracking corresponds to values TFE = 1 and TME = 1 for both measures; TFE > 1 implies that some GT tracks were fragmented, while TME > 1 indicates that some ST were incorrectly merged.

When using Eq. (2) to evaluate TME, different tracking algorithms cannot be objectively compared, because they will not only produce different numbers of track associations $(nST \sim GT)$, but also different total numbers of tracks ($nST$). For instance, when two track fragments detected by algorithm 1 are correctly connected by algorithm 2, both $nST \sim GT$ and $nST$ decrease. This increases the TME, even though no additional false connections occur (see Fig. S1).

To compensate for the decrease in $nST$ in a more accurate algorithm, we introduce a correction factor $\,{\rm{\Delta }}ST=nS{T}_{ref}-nST$, where $nS{T}_{ref}$ is the number of ST detected by the reference algorithm (algorithm 1), and $nST$ is the number of ST detected by the algorithm for which the merging error is being computed (algorithm 1 or 2). The corrected TME is computed as follows:

3 $$TM{E}_{corr}=\frac{nST \sim GT+{\rm{\Delta }}ST}{nST+{\rm{\Delta }}ST}=\frac{nST \sim GT+nS{T}_{ref}-nST}{nS{T}_{ref}}.$$

Due to this correction, the difference between $TM{E}_{corr}$ of two algorithms will be determined by the numerator alone. The denominator remains unchanged, because it equals the number of tracks detected by algorithm 1 ($nS{T}_{ref}$), independently of the algorithm for which the error is being computed. If algorithm 2 correctly connects two track fragments that are ignored by algorithm 1, both $nST$ and $nST \sim GT\,$decrease by 1, which results in both the numerator and $TM{E}_{corr}$ staying constant. On the other hand, a false connection does not decrease $\,nST \sim GT$, but only decreases $nST$ and hence both the numerator and the value of $TM{E}_{corr}$ do increase (Fig. S1).

For conciseness, we use the abbreviation TME in what follows to refer to $TM{E}_{corr}$ that is computed according to Eq. (3).

### Time-lapse microscopy videos

To evaluate the tracking performance of different versions of AMIT, we utilize the three previously recorded time-lapse microscopy videos of migrating polymorphonuclear neutrophils (PMN)^9^, as well as six videos that were newly generated following the same experimental protocol^9^. In short, PMN were isolated from human venous blood of healthy volunteers after informed consent by density gradient centrifugation using Polymorphprep (Fresenius Kabi Norge AS)^16^. Purity of PMN preparations was evaluated by flow cytometry using an anti-CD66b antibody (BD Bioscience) and only PMN with a purity of at least 95% were used for live cell imaging experiments. All protocols were approved by the Ethics Committee of the University Hospital Jena (permit number: 273-12/09).

A total of 2 × 10^5^ PMN was added into a µGrid cell culture imaging dish (ibidi GmbH) containing a total volume of 2 ml of RPMI1640 with 5% heat-inactivated human serum and 2.5 ng ml^−1^ of propidium iodide (PI, Sigma). PI was added to assess and visualize the viability of PMN throughout live-cell imaging. The dye is excluded by viable cells but can penetrate cell membranes of dying or dead cells and, therefore, was used to identify the rarely occurring dead cells that were not further considered in this analysis. PI is excited at 488 nm and emits at a maximum wavelength of 617 nm. Cells were incubated in an environmental control chamber at 37 °C and 5% CO_2_. Images were taken at six frames per minute over a period of at least one hour with both bright-field and fluorescence illumination using a LSM 780 confocal microscope which was focused on the bottom of the imaging dish (20x microscope objective Plan-APOCHROMAT 20x/0.8 NA). To evaluate the tracking performance and compute the track length, all videos were cropped to the first hour of the imaging period.

To test the algorithm’s performance in detecting cell-cell interactions, we also analyzed one time-lapse microscopy video of a confrontation assay, where migrating PMN were confronted with *Candida glabrata* cells. The video was used previously to develop and validate AMIT-v1 and the details of the experimental procedure are described elsewhere^2,10^.

## Results

We visually examined the tracking results of our previously developed AMIT-v1 framework and identified three possible reasons for incorrect track recognition. First, incorrect track matching is caused by the complex cell interactions, such as forming very dense clusters with many cells that may occasionally even move on top of each other. The second source of track fragmentation is inherently associated with the non-rigid nature of immune cells, which dynamically change their shape while migrating and interacting with other cells. The strength of AMIT-v1 is being able to segment non-rigid cells based on spatial and temporal variations in image intensities, but when these variations become too small during transient spreading of cells (Fig. 1b), the cell track gets interrupted. The third source of track fragmentation is associated with the gap size parameter. This parameter defines the number of time steps that are allowed to be missing when connecting two tracklets. When the gap size parameter is too low, only a few tracklets can be connected in this way, while a too high value of the gap size may result in false track merging.

In the following, we investigate how these three issues influence tracking performance. We demonstrate that the enhanced second version of AMIT (AMIT-v2) reduces track fragmentation and improves recognition of tracks as a whole. To objectively compare the tracking performance, we use a manually acquired ground truth, and we focus on migration assays of PMN, as these represent a benchmarking example for non-rigid cells.

### Higher cell numbers increase tracking errors

**Figure 3:**
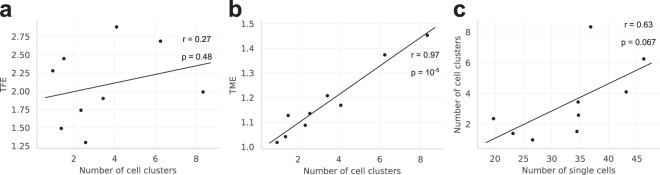
Tracking errors of AMIT-v1 in relation to the number of cells and cell clusters in the field of view. (**a**) The track fragmentation error (TFE) does not significantly increase with the number of cell clusters; (**b**) the track merging error (TME) is highly correlated with the number of cell clusters; (**c**) the number of cell clusters increases with the number of single cells in the field of view. The numbers of cells and cell clusters in the field of view are computed as the average over all time frames for each of the nine videos. The parameters r and p denote the Pearson correlation coefficient and the p-value for the Pearson correlation coefficient, respectively.

First, we examined how the number of cells and cell clusters in a field of view impact on the tracking performance measures, i.e. TFE and TME (Eqs (1, 2)). Analyzing nine videos with AMIT-v1 showed that both the TFE and TME slightly increased with the number of single cells (Fig. S2a,b), while the number of cell clusters affected the TFE and TME to a different extent (Fig. 3a,b). The increase of the TFE with the number of cell clusters was almost negligible (Fig. 3a), which implies that this error results mostly from the other two factors: missing spreading cells and inadequate gap size. On the other hand, the TME was found to be highly correlated with the number of cell clusters (Fig. 3b), which implies that the number of cell clusters is the major factor contributing to this error.

We further examined how the number of cell clusters depends on the number of cells in the field of view. To estimate the total number of cells we computed the number of single cells in the field of view averaged over all time frames. Cells forming clusters were relatively few and were neglected. Analyzing all nine videos with AMIT-v1 showed that the average number of cell clusters increases with the number of single cells (Fig. 3c). We used the same number of cells in all assays and the observed variations of the number of cells are due to a randomly selected field of view. Thus, if higher seeding densities are used, even higher numbers of cells will occur in the field of view and will likely promote cell clustering and increase tracking errors. To prevent this, one should keep cell densities in migration and confrontation assays as low as possible.

### Detection of spreading cells decreases track fragmentation error

**Figure 4:**
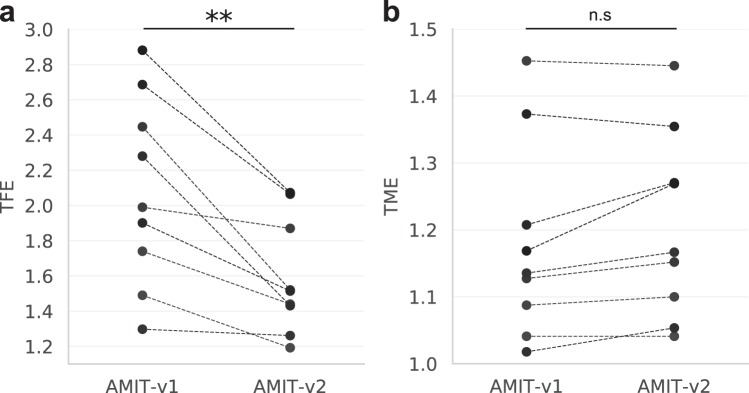
Tracking errors of AMIT-v1 and AMITv2 for the nine videos: (**a**) The track fragmentation error (TFE) significantly decreases due to the detection of spreading cells by AMIT-v2, p = 0.004 (Wilcoxon signed-rank test, n = 9, two-tailed); (**b**) the track merging error (TME) does not significantly change, p = 0.06 (Wilcoxon signed-rank test, n = 9, two-tailed).

Next, we evaluated the impact of spreading cells on track fragmentation. We applied AMIT-v1 and AMIT-v2 to the nine videos and used the default value for the gap size parameter, i.e. the gap size of three time frames^9^. The detection of spreading cells with AMIT-v2 significantly decreased the TFE relative to AMIT-v1 (Fig. 4a), which confirmed that an accurate segmentation of low-contrast cells is crucial to connect many track fragments (Fig. S3).

To confirm that the tracklet connection did not increase merging errors, we computed the TME with Eq. (3) to compare AMIT-v1 with AMIT-v2. Here, AMIT-v1 served as the reference algorithm, i.e. $nS{T}_{ref}$ corresponded to the number of system tracks generated by AMIT-v1. We found that, indeed, TME did not significantly increase in AMIT-v2 (Fig. 4b), which means that detecting spreading cells in AMIT-v2 reduced overall tracking errors.

### Higher gap sizes reduce the track fragmentation error

We further tried to improve tracking performance by adjusting the gap size parameter, and therefore examined how different gap size values influence TFE and TME. We computed TFE and TME according to Eqs (1, 3) for gap sizes ranging from zero to nine time frames. Because gap size zero yielded the highest number of tracklets, we used $nST$ detected with this value as the reference ($nS{T}_{ref}$).

**Figure 5:**
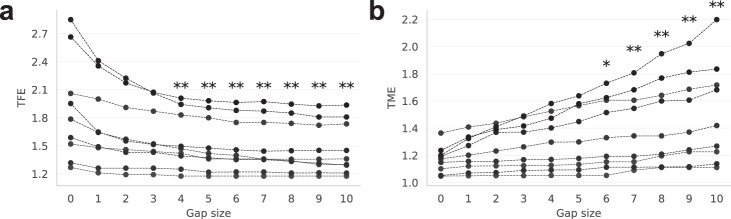
Tracking errors of AMIT-v2 for different values of the gap size parameter. (**a**) The track fragmentation errors (TFE) and (**b**) track merging errors (TME) for the nine videos. Stars indicate a significant difference with respect to gap size 3 (default value): ^*^p < 0.05, ^**^p < 0.01 (Wilcoxon signed-rank test, n = 9, two-tailed).

As expected, higher gap sizes reduced TFE and simultaneously increased TME (Figs 5 and S4). To examine, whether these changes were significant, we statistically compared the TFE and TME between the current default gap size value of three time frames and higher gap sizes. We found that a gap size value of four time frames and higher significantly reduced the TFE (Fig. 5a), while the TME significantly increased starting from the gap size values of six time frames and higher. This means that increasing the gap size from the current default value of three time frames to the value of five time frames does not significantly increase the merging error, but yields a decrease in the track fragmentation error.

It should be noted that optimizing the gap size in the way presented here actually requires a ground truth, e.g. by a manual tracking, which would imply that the task has already been solved before automated analysis is done. Therefore, for any practical application, we need to estimate the optimal gap size by other means.

### The optimal gap size can be estimated from cell size and speed

We further demonstrate that the optimal gap size can be estimated without computing the performance measures TFE and TME. Applicable values of the gap size depend on the frame rate of the microscopy video and the typical cell size and speed.

When we connect two cells separated by a time gap of $g$ frames, this corresponds to a time interval of $(g+1){\rm{\Delta }}t$, where ${\rm{\Delta }}t$ is the time interval between two consecutive time frames. During this time, a cell travels a distance $\,d\,=\,(g+1)v{\rm{\Delta }}t$, where $v$ represents an estimation for the speed of the cells. For high values of the gap size, $d$ becomes so large that tracklets cannot be unambiguously connected: this will increase TME. While on the one hand we aim to maximize the gap size to reduce TFE, on the other hand the gap size should not be larger than a certain critical value that permits a cell to travel a critical distance $\,d$:

4 $$g=\frac{d}{v{\rm{\Delta }}t}-1.$$

This equation can be used to estimate the optimal gap size for an arbitrary video data once values for $v$ and $\,d$ have been determined. We found that the best agreement with the gap size values estimated from TFE and TME (Fig. 5) was provided by $v$ equal to the average cell speed and $d$ equal to the average cell diameter. Using these values to compute the optimal gap size for all nine videos yielded gap size values between three and six time frames (Fig. S5). Averaging over all nine videos yielded the mean value 4.6 ± 1.3 for the optimal gap size, i.e. a rounded value of five time frames. Interestingly, increasing the gap size from three to five also did not significantly increase TME (Fig. 5). Therefore, we come to the conclusion that raising the gap size value from three to five time frames will reduce track fragmentation without increasing merging errors.

### Recovered spreading cells and optimized gap size yield longer cell tracks

**Figure 6:**
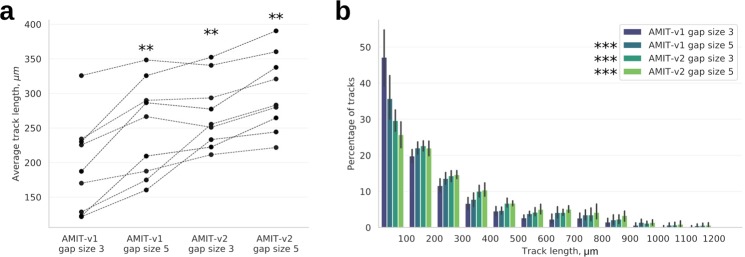
Track lengths for AMIT-v1 and AMIT-v2 with gap sizes of three and five time frames. (**a**) The average track length; stars indicate significant differences with respect to AMIT-v1 with gap size 3: ^**^p < 0.01 (Wilcoxon signed-rank test, n = 9, two-tailed); (**b**) distribution of tracks with different lengths; stars indicate significant differences with respect to AMIT-v1 with gap size 3: ^***^p < 10^−9^ (Kolmogorov–Smirnov test, two-tailed).

We so far showed that detecting spreading cells and increasing gap size reduced TFE (see Figs 4, 5). Next, we evaluated whether the drop in TFE is correlated with our main objective of detecting longer cell tracks. We applied AMIT-v1 and AMIT-v2 with gap sizes three and five time frames to nine videos of migrating PMN. We found that including the tracks of spreading cells in AMIT-v2 increased the average track length by 7–71%, which in combination with a higher gap size made the cell tracks 11–120% longer (see Fig. 6a) and allowed to follow cells for up to 3 hours when analyzing videos of extended length (Fig. S6). This dramatic increase in track length was mainly due to connecting or prolonging very short tracks, whose numbers have dropped after applying AMIT-v2 (see Figs 6b and S6). While both detection of spreading cells and increasing the gap size yielded significantly longer cell tracks, the longest tracks were recognized by a combination of these two improvements (see Fig. 6).

### Longer cell tracks provide longer records of cell-cell interactions

**Figure 7:**
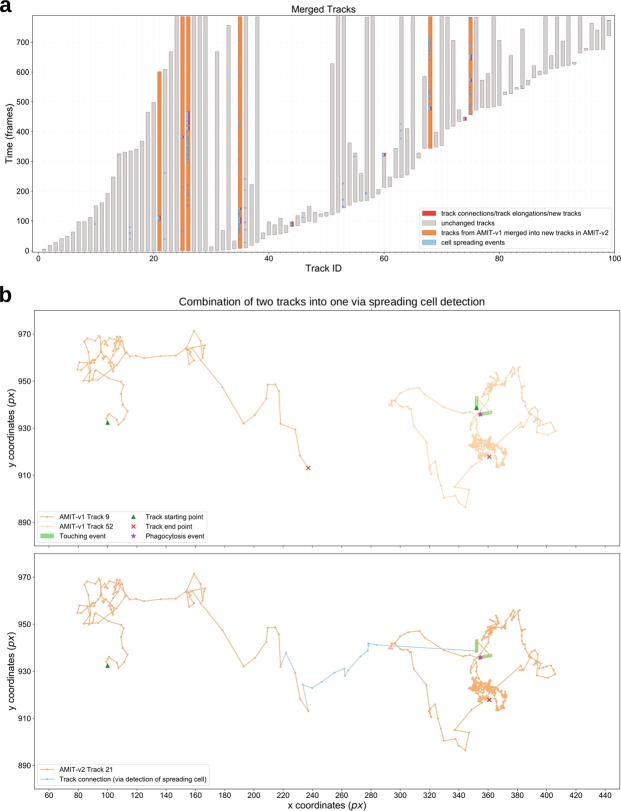
Comparative tracking results of AMIT-v1 and AMIT-v2 (both with a gap size of five time frames) applied to a confrontation assay of PMN with Candida glabrata. (**a**) PMN tracks generated by AMIT-v2 are shown as a straight gray bar arranged by the starting time point in the video. PMN tracks from AMIT-v1 that were merged in AMIT-v2 are highlighted in orange. (**b**) Exemplary PMN track that was fragmented in AMIT-v1 (top) but was merged in AMIT-v2 (bottom). Video frame rate is six frames per minute, pixel size is 0.2 µm.

To demonstrate the impact of enhanced track recognition on the record of cell-cell interactions, we applied both versions of the algorithm with gap size five time frames to a one hour video of a confrontation assay (Fig. S7), where PMN were confronted with the human-pathogenic fungus *Candida glabrata*. Figure 7a shows the PMN tracks recognized by AMIT-v2 and arranged by their starting time point in the video. The tracks from AMIT-v1 that were merged by AMIT-v2 are highlighted in orange. In Fig. 7b, we present a detailed example of such a merged track detected by AMIT-v2: this particular PMN had no fungal contact for the first 117 time frames of the video, after which it touched a *Candida* cell two times, and then phagocytosed the fungus. In contrast, when tracking with AMIT-v1, this time-ordering of cell interaction events was lost, because the track of the PMN was interrupted and erroneously assigned to two separate shorter tracks. Thus, due to enhanced recognition of tracks as a whole, AMIT-v2 enables investigating correlations and potentially cause-effect relations between various interaction events.

## Discussion

In this study, we identified three sources of track fragmentation errors during automatic tracking: high cell densities, low contrast cells, and inadequate gap size. High cell densities lead to frequent cell-cell contacts that appear as transient clusters in the acquired images, which inevitably results in tracklet mismatching when trying to resolve these clusters and increases not only track fragmentation but also track merging errors. An accurate cluster splitting is challenging and sometimes impossible to achieve even by an extensive algorithm optimization. In fact, even a manual tracking often fails to correctly assign cells in dense clusters to their corresponding tracks. On the other hand, manually tracked ground truth data is necessary to evaluate the performance of automated tracking algorithms, and when such ground truth contains mistakes and ambiguities, the algorithmic solutions cannot be adequately validated. Thus, tracklet mismatching due to cell clustering is difficult to rule out, other than by keeping the cell densities in migration and confrontation assays as low as possible.

In contrast to high cell densities, the other two sources of track fragmentation can be addressed by improving the automated tracking algorithm. We therefore implemented an optimized version of our previously published AMIT framework, which enhances segmentation of low-contrast cells and uses an optimized value of the gap size to improve combination of tracklets and enhance recognition of whole cell tracks.

By solving the issue of segmenting low contrast cells, we managed to significantly reduce track fragmentation errors without increasing track merging errors (see Fig. 4). These results imply that improving segmentation of low contrast spreading cells is an efficient way to enhance the accuracy of label-free tracking, which is highly beneficial for studying migration and interactions of human cells. Being able to identify low-contrast cells is especially relevant when tracking immune cells, because transient spreading is a common phenotype that immune cells adopt during activation and cell-cell interactions^12,13^.

We managed to further decrease track fragmentation by optimizing the gap size parameter from the original default value of three time frames to the value of five time frames. This value for the optimal gap size could be estimated based on the maximum cell diameter, average cell speed and the frame rate of the microscopy videos according to Eq. 4. The proposed equation may be of general use in cell tracking, because many algorithms use similar strategies for connecting track fragments.

Addressing two out of three sources of track fragmentation in AMIT-v2 resulted in the detection of longer cell tracks. We managed to increase the average length of detected tracks by up to 120% and to follow individual cells through a time span of up to three hours (see Fig. S6). As we have demonstrated by the study of a confrontation assay for PMN and *C. glabrata*, longer cell tracks provide a longer history of interactions that a cell experienced during the course of the study (see Figs 7 and S7). Such comprehensive record allows to identify rare events^3^, or find patterns and correlations in cell behavior, e.g. to relate the probability of phagocytosis to the number of preceding interactions with other cells and thus to elucidate mechanisms of immune cell activation towards phagocytosis of pathogens. Cell tracks of highest possible length are also needed to identify complex migration patterns, such as switches in migration mode^17,18^. Moreover, a joint analysis of interaction and migration patterns may help to answer the question of whether the changes in migration mode are caused by interactions with other cells. Answering these and related questions about cell migration – based on the recognition of cell tracks as a whole – will in the long run advance our understanding of immune cell function in health and disease.

## Supplementary information

Supplementary Info

## Data Availability

The authors declare that all relevant data supporting the findings of this study are available within the paper (and its Supplementary information file). Any raw data can be downloaded from https://asbdata.hki-jena.de/AlZabenEtAl2018_SciRep/.
