# Supplementary material for: Automated tracking of label-free cells with enhanced recognition of whole tracks
Source: Sci Rep. 2019 Mar 1;9:3317. doi: 10.1038/s41598-019-39725-x (PMC6397148; doi:10.1038/s41598-019-39725-x)
Supplement: Supplementary file 1 — Supplementary Info [file 41598_2019_39725_MOESM1_ESM.pdf]

Supplementary Information

Automated tracking of label-free cells with enhanced recognition of whole tracks

Naim Al-Zaben[1,2,†], Anna Medyukhina[1,†], Stefanie Dietrich[1,2], Alessandra Marolda[2,3], Kerstin Hünniger[3,4], Oliver Kurzai[3,4,5], Marc Thilo Figge[1,2,5,*]

[1]Applied Systems Biology, Leibniz Institute for Natural Product Research and Infection Biology – Hans Knöll Institute (HKI), Jena, Germany.
[2]Faculty of Biological Sciences, Friedrich Schiller University Jena, Jena, Germany.
[3]Fungal Septomics, Leibniz Institute for Natural Product Research and Infection Biology – Hans Knöll Institute (HKI), Jena, Germany.
[4]Institute of Hygiene and Microbiology, University of Würzburg, Würzburg, Germany.
[5]Center for Sepsis Control and Care (CSCC), Jena University Hospital, Jena, Germany.

† These authors contributed equally to this work.

* Correspondence should be addressed to M.T.F.: thilo.figge@leibniz-hki.de

FIGURE S1:

Case 1
correct connection

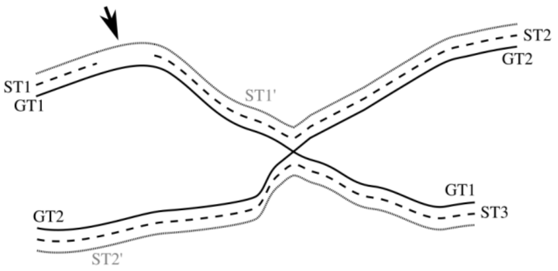

Case 2
false connection

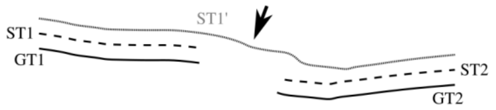

Case 1:

	algorithm 1	algorithm 2
nGT	2	2
nST	3	2
nST~GT	5 (ST1~GT1, ST2~GT1, ST2~GT2, ST3~GT2, ST3~GT1)	4 (ST1~GT1, ST1~GT2, ST2~GT2, ST2~GT1)
ST_{ref}	3	3
dST	0	1
TFE	5 / 2 = 2.5	4 / 2 = 2
TME	5 / 3 = 1.67	4 / 2 = 2
TME_{corr}	(5 + 0) / (3 + 0) = 1.67	(4 + 1) / (2 + 1) = 1.67

Case 2:

	algorithm 1	algorithm 2
nGT	2	2
nST	2	1
nST-GT	2 (ST1~GT1, ST2~GT2)	2 (ST1~GT1, ST1~GT2)
ST_{ref}	2	2
dST	0	1
TFE	2 / 2 = 1	2 / 2 = 1
TME	2 / 2 = 1	2 / 1 = 2
TME_{corr}	(2 + 0) / (2 + 0) = 1	(2 + 1) / (1 + 1) = 1.5

$$TFE = \frac{nST{\sim}GT}{nGT} \qquad TME = \frac{nST{\sim}GT}{nST} \qquad TME_{corr} = \frac{nST{\sim}GT + dST}{nST + dST} \qquad dST = ST_{ref} - ST$$

Figure S1. Exemplary computation of track fragmentation error (TFE), track merging error (TME) and corrected track merging error (TME_{corr}) for two arbitrary algorithms, where the second algorithm generates an additional track connection (arrow) that is either correct or wrong.

FIGURE S2:

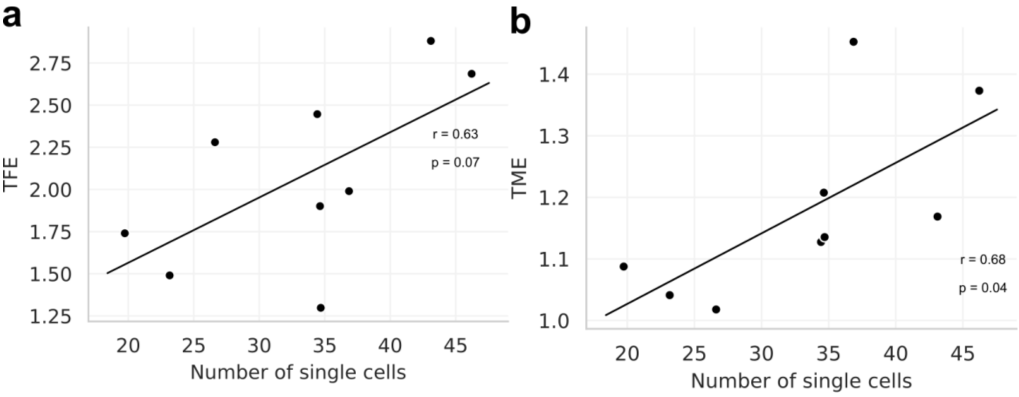

Figure S2. The track fragmentation error (TFE) (a) and the track merging error (TME) (b) of AMIT-v1 increase with the average number of single cells in the field of view. The parameters r and p denote the Pearson correlation coefficient and the p-value for the Pearson correlation coefficient, respectively.

FIGURE S3:

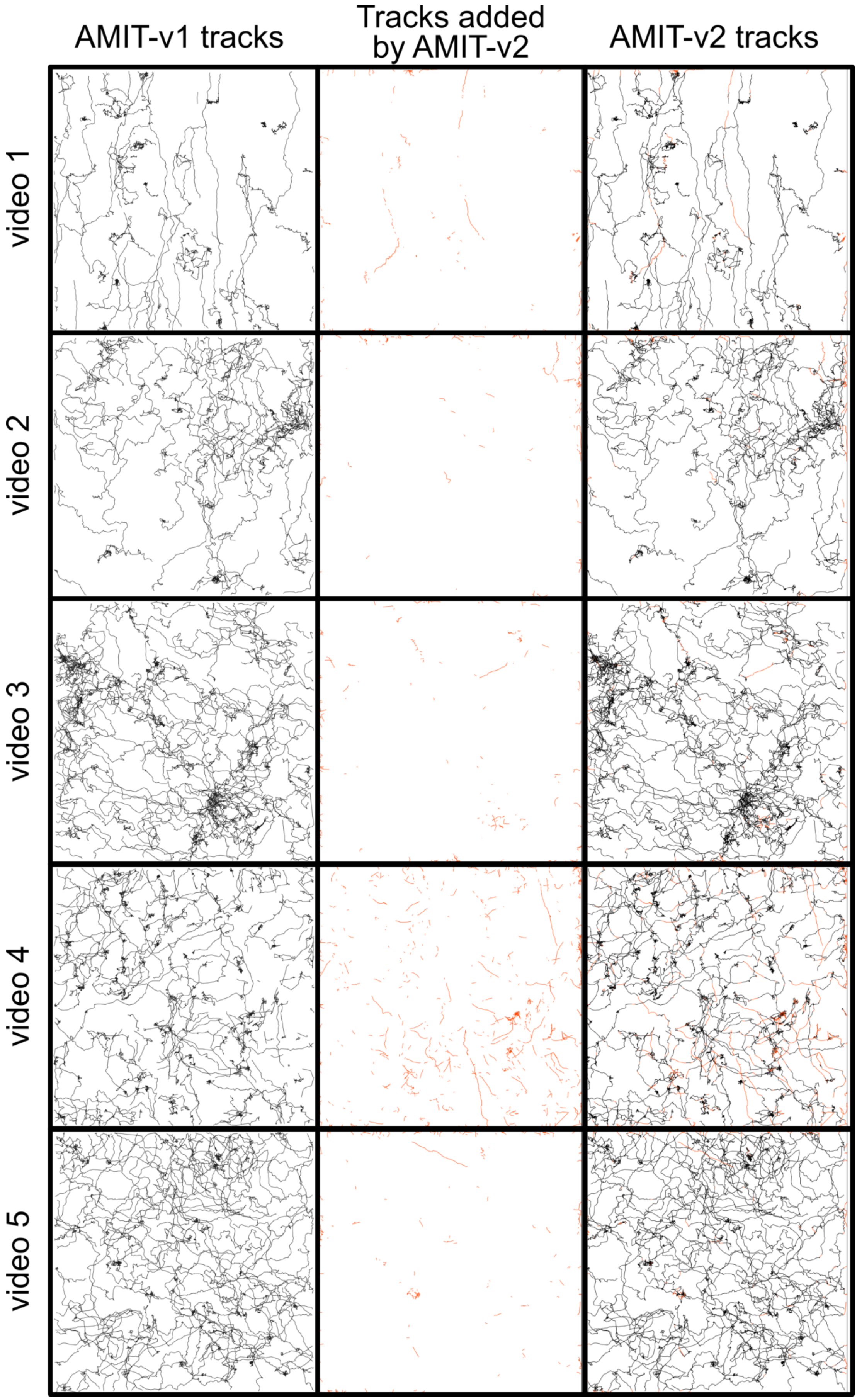
AMIT-v1 tracks
Tracks added by AMIT-v2
AMIT-v2 tracks
video 1
video 2
video 3
video 4
video 5

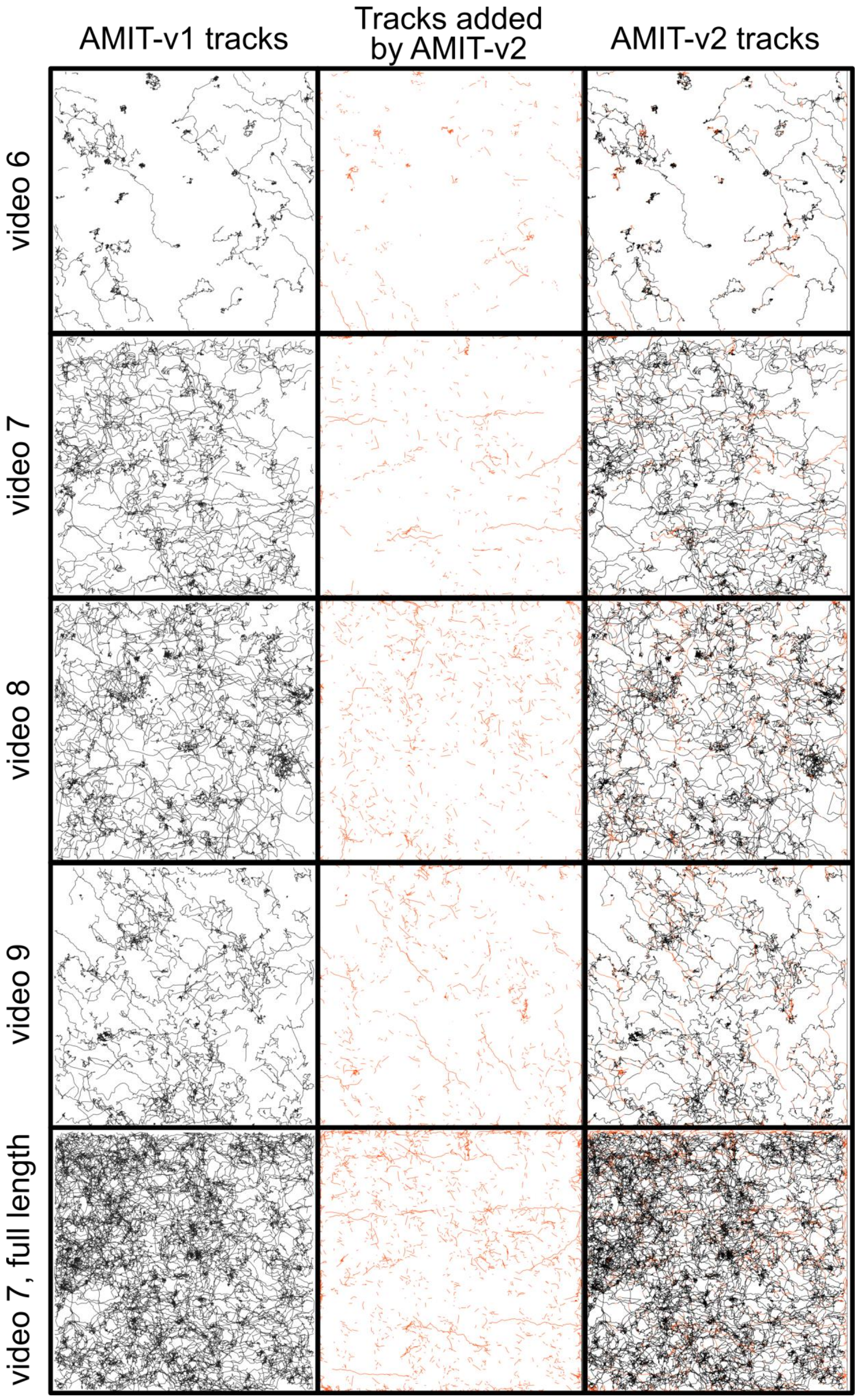

Figure S3. Final tracks generated by AMIT-v1 and AMIT-v2. The size of the field of view is 425× 425 µm.

FIGURE S4:

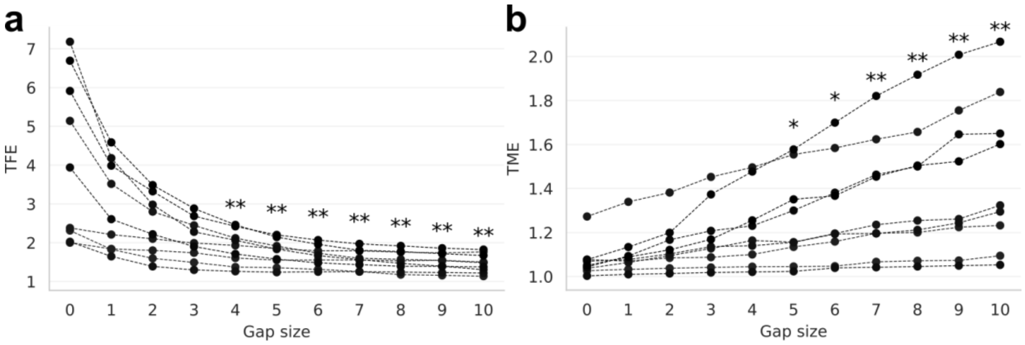

*Figure S4. Tracking errors of AMIT-v1 for different values of the gap size parameter. (a) Track fragmentation errors (TFE); (b) track merging errors (TME). Stars indicate significant difference with respect to gap size 3: * $p < 0.05$, ** $p < 0.01$ (Wilcoxon signed-rank test, n=9, two-tailed).*

FIGURE S5:

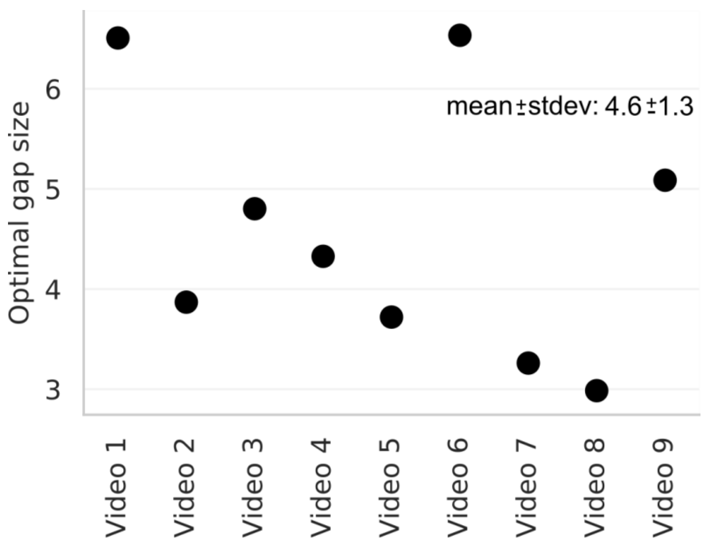

Figure S5. Optimal gap size computed according to equation (4) for videos 1-9.

FIGURE S6:

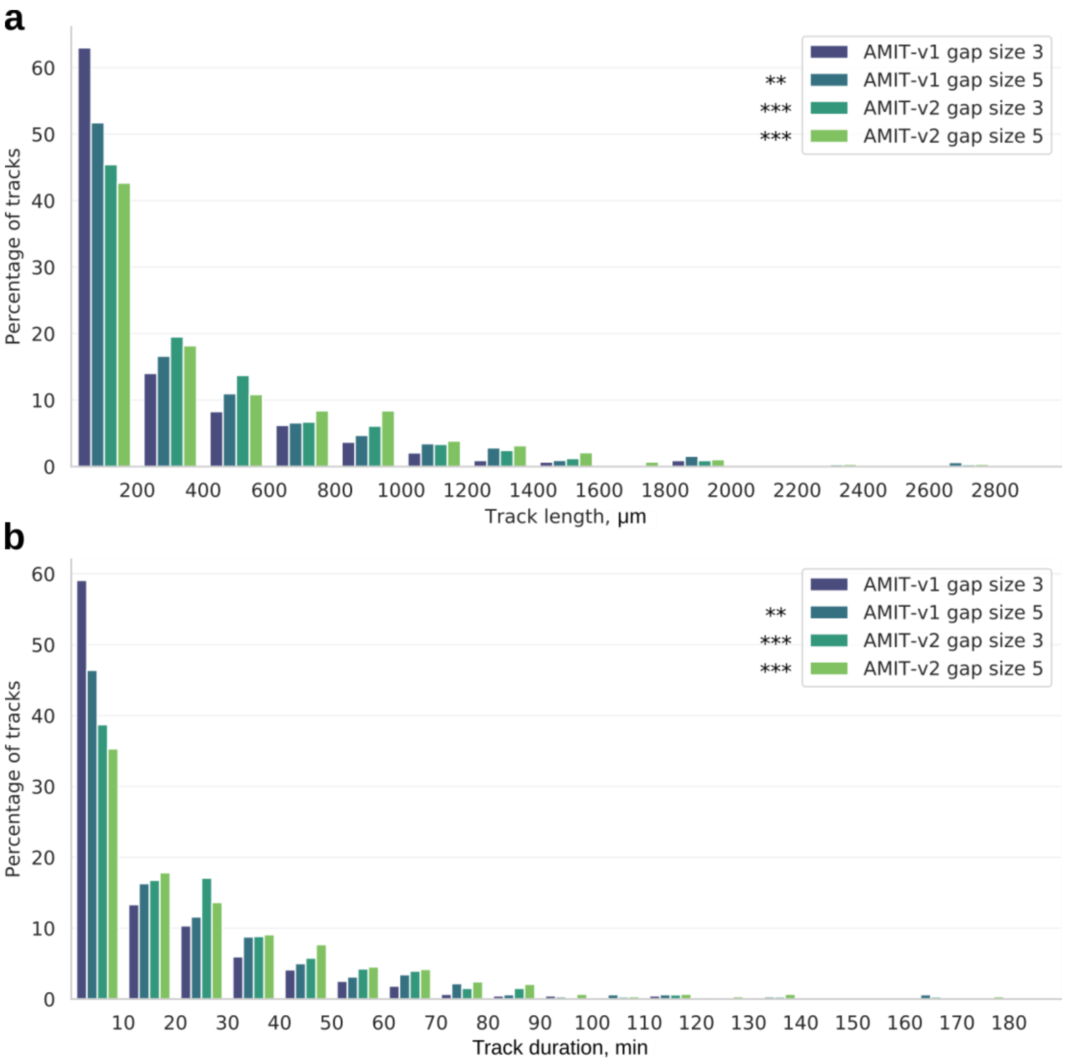

*Figure S6. Distribution of tracks with different (a) track lengths and (b) track durations in a full-length video of three hours for AMIT-v1 and AMIT-v2 with gap sizes three and five time frames, stars indicate significant difference with respect to AMIT-v1 with gap size 3: ** $p < 0.01$, *** $p < 10^{-5}$ (Kolmogorov–Smirnov test, two-tailed).*

FIGURE S7:

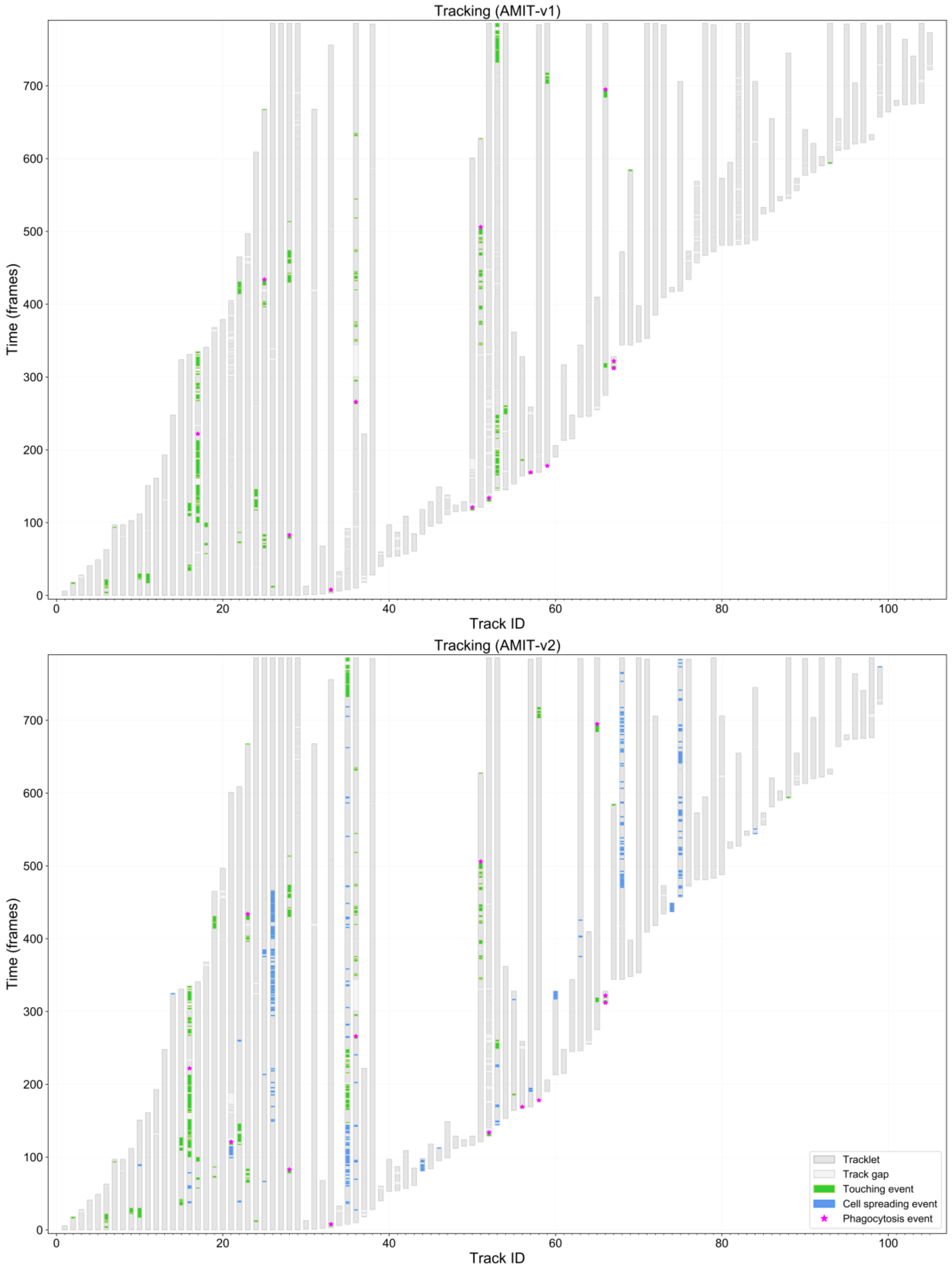

Figure S7. Tracks of polymorphonuclear neutrophils (PMN) confronted with Candida glabrata generated by AMIT-v1 (top) and AMIT-v2 (bottom) arranged by their starting time point in the video; touching events are highlighted in green, phagocytosis events are indicated by stars. Frame rate is six frames per minute.
